# Simulation versus real-world performance: a direct comparison of emergency medicine resident resuscitation entrustment scoring

**DOI:** 10.1186/s41077-019-0099-4

**Published:** 2019-05-01

**Authors:** Kristen Weersink, Andrew K. Hall, Jessica Rich, Adam Szulewski, J. Damon Dagnone

**Affiliations:** 1 Department of Emergency Medicine, Queen’s University, Kingston Health Sciences Center c/o 76 Stuart St, Kingston, ON K7L2V7 Canada; 2 Faculty of Education, Queen’s University, Kingston, ON Canada

## Abstract

**Background:**

Simulation is increasingly being used in postgraduate medical education as an opportunity for competency assessment. However, there is limited direct evidence that supports performance in the simulation lab as a surrogate of workplace-based clinical performance for non-procedural tasks such as resuscitation in the emergency department (ED). We sought to directly compare entrustment scoring of resident performance in the simulation environment to clinical performance in the ED.

**Methods:**

The resuscitation assessment tool (RAT) was derived from the previously implemented and studied Queen’s simulation assessment tool (QSAT) via a modified expert review process. The RAT uses an anchored global assessment scale to generate an entrustment score and narrative comments. Emergency medicine (EM) residents were assessed using the RAT on cases in simulation-based examinations and in the ED during resuscitation cases from July 2016 to June 2017. Resident mean entrustment scores were compared using Pearson’s correlation coefficient to determine the relationship between entrustment in simulation cases and in the ED. Inductive thematic analysis of written commentary was conducted to compare workplace-based with simulation-based feedback.

**Results:**

There was a moderate, positive correlation found between mean entrustment scores in the simulated and workplace-based settings, which was statistically significant (*r* = 0.630, *n* = 17, *p* < 0.01). Further, qualitative analysis demonstrated overall management and leadership themes were more common narratives in the workplace, while more specific task-based feedback predominated in the simulation-based assessment. Both workplace-based and simulation-based narratives frequently commented on communication skills.

**Conclusions:**

In this single-center study with a limited sample size, assessment of residents using entrustment scoring in simulation settings was demonstrated to have a moderate positive correlation with assessment of resuscitation competence in the workplace. This study suggests that resuscitation performance in simulation settings may be an indicator of competence in the clinical setting. However, multiple factors contribute to this complicated and imperfect relationship. It is imperative to consider narrative comments in supporting the rationale for numerical entrustment scores in both settings and to include both simulation and workplace-based assessment in high-stakes decisions of progression.

**Electronic supplementary material:**

The online version of this article (10.1186/s41077-019-0099-4) contains supplementary material, which is available to authorized users.

## Background

Acute care physicians are often faced with critical time-sensitive decisions in the resuscitation setting. Assessment of competence in this complex clinical environment is fraught with bias, poor reliability, and practical difficulty [1]. From the perspective of those training and certifying physicians, simulation is becoming an attractive option for assessing physician competence in certain domains [2, 3], but it is still unclear if competence demonstrated in the simulation setting can be used as a valid indicator of competence in the clinical setting [4].

The body of validity evidence supporting simulation as a performance-based environment for assessment is constantly growing [5]. There is evidence that simulation-based learning and assessment are effective in increasing medical expert knowledge [6], procedural skills [7, 8], learner confidence for real-life practice, discriminating the novice from expert learner [9], and improving patient outcomes [4, 10]. Activity patterns of physicians in clinical scenarios have been shown to be similar in both the simulated and real environment [11], and acute care team performance in both settings has been shown to be similar as well [12]. Furthermore, there is evidence that simulation-based assessment outcomes correlate with residents’ scores on oral examinations [13] and portfolio-based assessment scores of medical expert and communication domains on in-training evaluation reports [14]. What is missing is an understanding of the relationship between simulation performance and workplace-based clinical competence in more multifarious tasks such as resuscitation. There is a paucity of research in this area, with most studies focused on procedural tasks with limitations of small and biased sampling of subjects, incomplete reporting of methodology, and limited applicability outside of a particular simulation model or technical skill [15–18].

The continued focus on patient-centered care and the more recent transition to competency-based medical education (CBME) in postgraduate training programs both lend themselves to increased use of simulation for learning *and* assessment. Current written and oral examinations test the “knows” and “knows how” components of Miller’s pyramid [19], a framework for assessing clinical competence in medical education. Simulation-based training expands learning and assessment opportunities to include “shows how” in an environment where residents can safely practice and receive feedback on essential clinical skills [20]. Furthermore, standardized workplace-based assessments are difficult to implement due to the variability of clinical encounters. This is a hurdle that can be overcome by simulation-based assessment [21]. Demonstration of competence in managing critical but rare situations––a necessary task to ensure patient safety––may in fact *only* be accomplished in simulation environments.

Assessment in CBME typically focuses on entrustment scoring, a method that has been shown to improve reliability compared to more traditional checklist methods [22, 23]. Entrustment, or the judgment of a trainee’s readiness to provide care under decreasing levels of supervision [24], is a tacit concept that is already intuitively utilized by supervising physicians every day in clinical practice. Thus, the use of entrustment scales for making global assessments of workplace-based performance typically resonates with front-line faculty [1]. Using an entrustment scoring system in the simulation environment may allow for interpretation and extrapolation to various clinical scenarios in the workplace.

The aim of the current study was to test the inference of extrapolation within Kane’s validity framework [25], through direct comparison of simulation and workplace-based clinical performance in the resuscitation of the critically ill. Kane’s framework argues for four inferences of validity: scoring, generalizability, extrapolation, and implications [25]. There is already a strong argument for the validity of simulation as an assessment opportunity with respect to the inferences of scoring and generalizability [3, 26, 27]. Extrapolation takes the assessment from the “test-lab” to the “real-world” environment and can be evaluated in terms of distinguishing learner stages (i.e., compared to experts), or more accurately, in terms of the correlation between a test-environment to the real-world environment [25]. We hypothesized that there would be a moderate positive correlation between resident performance in the simulation setting and performance in the emergency department (ED) given the obvious differences between highly controlled simulation environments and uncontrolled workplace-based settings.

## Methods

### Setting and participants

A prospective cohort study of Queen’s emergency medicine (EM) residents was designed and approved by the Health Sciences and Affiliated Teaching Hospitals Research Ethics Board at the Queen’s University. All EM residents from postgraduate year (PGY) one to five enrolled at the Queen’s University from July 1, 2016 to June 30, 2017 (*n* = 28) were recruited for the study. The study was carried out at the Queen’s Clinical Simulation Center, Kingston General Hospital, and through online collaboration with expert raters from June 2016 to July 2017. Residents provided informed consent to participate in the study, including video recording of their performances in the simulation lab.

### QSAT modification to create the RAT

The Queen’s simulation assessment tool (QSAT) [27] was modified to create the entrustment-based resuscitation assessment tool (RAT) and subsequently used to directly compare EM residents’ performance in the simulation environment to performance in the ED. A strong validity argument for the QSAT has been previously published [27] along with comparisons of the QSAT to in-training evaluation report scoring [14] and the multicenter implementation of the QSAT [28]. However, limitations to the QSAT have been noted, including the need for scenario customization and a desire for the tool to utilize an entrustment-based global assessment score. Therefore, limited modifications to the QSAT (Additional file 1) were undertaken to create the workplace-based RAT. The two modifications were (1) the development of generic behavioral anchors for resuscitation performance using a modified Delphi process [29] for each domain (primary assessment, diagnostic actions, therapeutic actions and communication) and (2) the replacement of the global assessment scale with a contemporary entrustment scale [30]. A pilot study has demonstrated a strong correlation between the existing/original global assessment score of the QSAT and the chosen entrustment score [31].

A purposeful sample of practicing physicians in critical care, local EM faculty, external EM faculty, and junior and senior residents were chosen to participate in the derivation of anchors. Specific individuals were invited to participate based on past experience with the QSAT and qualifications reflecting expertise in EM and simulation-based education and assessment. An email invitation was sent out, explicitly stating that participation would require adherence to a revision timeline including three rounds of a modified Delphi via FluidSurveys™.

In the first survey, participants were asked in an open-ended format to generate behavioral anchors for each of the four domains of assessment of the current QSAT. The focus of assessment for the RAT was competence in resuscitation performance, as defined by an entrustable professional activity [32] written by study authors (AH, DD): “Resuscitate and manage the care of critically ill medical/surgical patients”. The anchors refer to critical component actions for successful resuscitation in the ED. The anchors were compiled by thematic analysis by researcher KW and reviewed by AH and JR, all blinded to participant identity.

In round two, the most frequently cited anchors for each domain were then distributed to the experts via a second survey. In this round, the same participants were asked to rank each anchor according to importance, based on a 5-item Likert scale (1 = not important, 5 = extremely important), and explain each ranking through an open response question. An inclusive list of important anchors for each assessment domain was used to generate the first draft of the complete RAT. The draft RAT was then distributed to the experts for a third round of minor revisions to ensure experts have reached agreement on the inclusion and wording of specific anchors.

Following derivation of the RAT, a multipronged approach to tool introduction and rater training was provided for all EM attending physicians and residents. The RAT was presented and described at departmental rounds, and faculty were trained in small groups in the ED while on shift by study investigators (AH, DD). Resident RAT training was provided as a special session within the core training curriculum early in the academic year (AH).

### Workplace-based resuscitation assessment and simulation-based resuscitation assessment

Residents were opportunistically assessed by their attending EM physician utilizing the RAT while on shift in the Kingston General Hospital ED. Resuscitation cases were defined as any case involving critical illness/injury that required life-threatening critical care, as described in detail by provincial fee codes [33], familiar to all EM physicians in Ontario. The decision to complete an assessment using the RAT was left to the discretion of the staff EM physician and the resident on shift. The clinical context of the case on which the RAT was completed was recorded on the RAT.

EM residents participated in simulation-based objective structured clinical examinations (OSCEs) in August 2016 and February 2017 as part of their established EM education program [34]. The OSCEs were held at the Queen’s Clinical Simulation Center. Each examination involved two previously developed and piloted resuscitation scenarios involving nurse and respiratory technologist actors [35]. The four cases assessed in the simulation-based OSCEs were set a priori and included a gastrointestinal bleed causing pulseless electrical activity cardiac arrest, chronic obstructive pulmonary disease exacerbation requiring intubation, ventricular fibrillation due to ST-elevation myocardial infarction, and hyperkalemia-induced bradycardia. In summary, each OSCE included two resuscitation cases, so a resident had the potential to be assessed on four cases, each with a single global entrustment score and opportunity to rationalize the numerical score with narrative feedback.

Resident performance was scored using the RAT by an in-person rater and video recorded. In order to measure the reliability of the scoring by the in-person rater, the video recorded performance was also scored by a blinded external rater using the RAT. In-person raters and external raters not involved in RAT development received an orientation training session in which they rated a standardized sample of training video recordings and reviewed with one of the investigators (AKH) until consensus scoring was achieved. Of note, some of the residents were invited to wear eye-tracking glasses during the OSCEs as part of a separate, unrelated study.

### Analysis

Mean entrustment scores were computed for each resident for the summer 2016 OSCE, winter 2017 OSCE, and workplace-based assessments. Scores were compared using the Pearson product-moment correlation coefficient to determine the linear relationship between mean entrustment scores on OSCE simulation-cases and on workplace-based assessments. To determine whether there was any difference in residents’ simulation performance on OSCE scores in the summer 2016 and the winter 2017, a paired-samples *t* test was conducted. Intraclass correlation coefficients, using a two-way random effects model with absolute agreement, were used to measure the interrater reliability between live and blind ratings of resident entrustment on the four OSCE cases. Residents with missing data (either no OSCE or no workplace-based data) were excluded from the analysis.

Narrative comments collected on the RAT for both workplace-based assessments and simulation-based assessments were coded using inductive thematic analysis [36]. Codes were identified and grouped into themes and then compared across simulation and workplace-based settings by author KW and subsequently reviewed by AH.

## Results

**Fig. 1:**
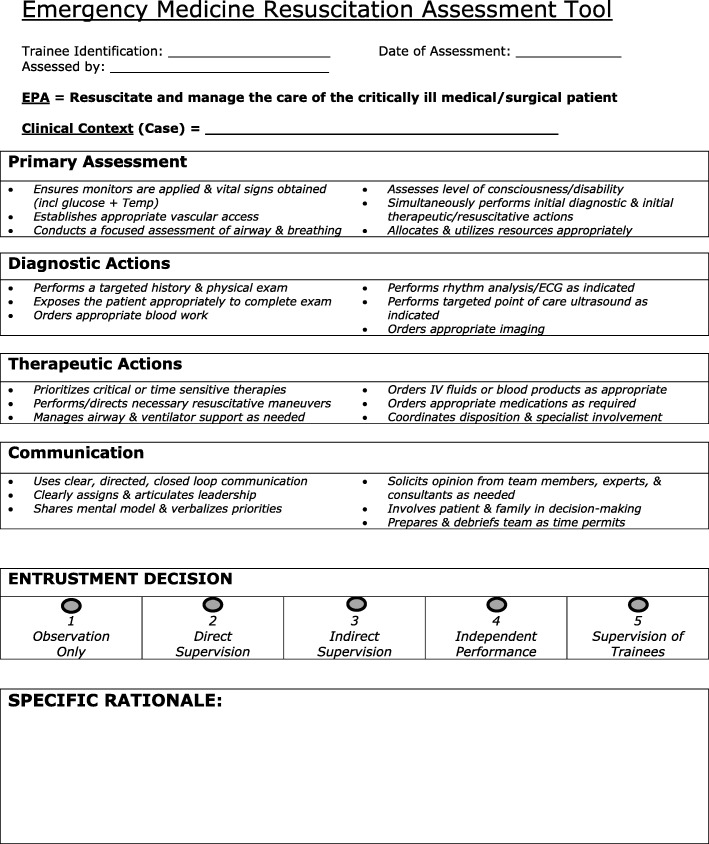
Workplace-based resuscitation assessment tool

The expert panel who engaged in our modified Delphi process consisted of eight resuscitation and medical education experts: one critical care Queen’s staff physician, two Queen’s EM residents (PGY2 and PGY4), and five staff EM physicians from the Queen’s University (*n* = 4) and the University of Toronto (*n* = 1). Six of the respondents had either advanced degrees in medical education or were fellowship trained in simulation. Compliance with the expert process and associated timeline was adhered to by all participants. The final version of the RAT is shown in Fig. 1.

Twenty-eight residents consented to their data being used in this study. However, upon review of the data, 11 of these residents were excluded due to insufficient workplace-based RAT or OSCE data. While participation in the OSCE was considered mandatory, residents who were away on rotation or vacation, or were ill, were excused from participating. As a result, some residents were assessed in one OSCE (two cases) or did not participate in an OSCE at all. Data from 17 residents (61%) were ultimately included in the analysis.

Of the 17 residents included in our sample, 14 residents participated in the summer 2016 OSCEs and 15 residents participated in the winter 2017 OSCEs. There were three PGY5, four PGY4, seven PGY3, two PGY2, and one PGY1 resident. All residents had a minimum of 10 h of experience in the simulation lab prior to assessment in the first OSCE. The number of workplace-based assessments completed for any one resident ranged from one to nine, with 88% of residents having completed at least two assessments. The clinical cases assessed in the workplace were heterogeneous, including cardiac arrest, respiratory failure, seizures, toxins, stroke, and pediatric resuscitation (see Table 1).

**Table 1:** OSCE and RAT cases July 1, 2016–June 30, 2017

OSCE		RAT	
Case	*n*	Case	*n*
GI bleed/PEA arrest	27	Cardiac arrest	23
COPDE	28	Other (sepsis, GI bleed, medical arrest)	15
VFib/STEMI	31	Respiratory failure	9
Bradycardia/hyperkalemia	32	Pediatric resuscitation	6
		Toxin/altered LOC	5
		Seizure	4
		Stroke/ICH	2

*OSCE* objective structured clinical exam, *RAT* resuscitation assessment tool, *GI* gastrointestinal, *PEA* pulseless electrical activity, *COPDE* chronic obstructive pulmonary disease exacerbation, *VFib* ventricular fibrillation, *STEMI* ST elevation myocardial infarction, *ICH* intracranial hemorrhage

**Fig. 2:**
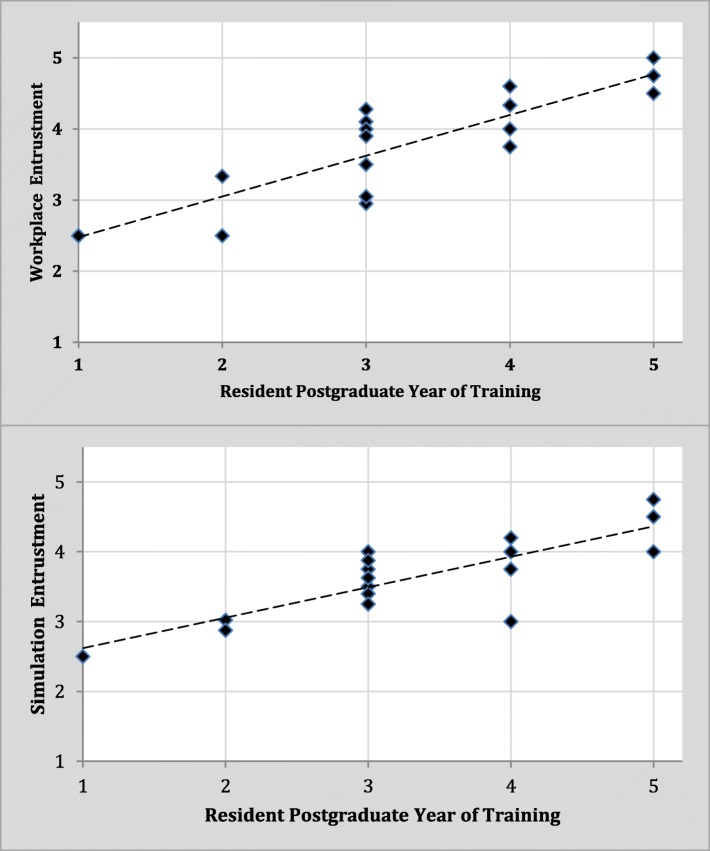
Scatterplot comparing mean entrustment scores from workplace-based assessment and simulation-based assessment with postgraduate year of training. Dashed line indicates trendline

**Fig. 3:**
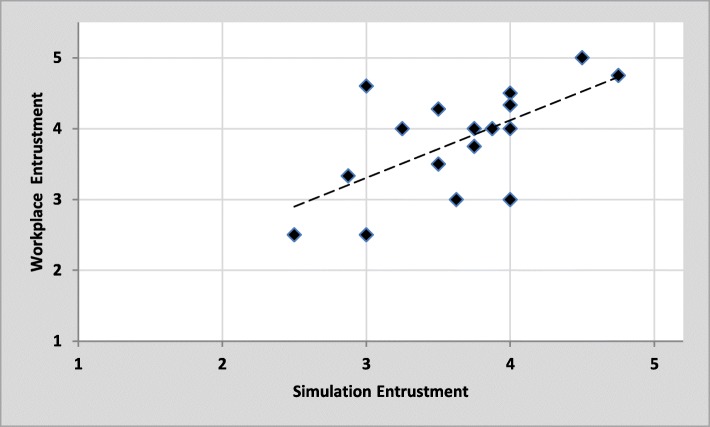
Scatterplot comparing mean entrustment scores in the simulated resuscitation vs. mean entrustment scores from workplace-based assessment for each resident (*N* = 17). Dashed line indicates trendline

Mean entrustment scores from workplace-based assessment and simulation-based assessments are plotted by PGY in Fig. 2. Mean entrustment scores in the simulated resuscitation OSCEs were compared with mean entrustment scores from workplace-based assessments for each resident in Fig. 3. A statistically significant moderate-positive correlation was found between mean entrustment scores in the simulated and workplace-based settings (*r* = 0.630, *n* = 17, *p* < 0.01). There was a statistically significant improvement in resident’s mean entrustment scores on simulated OSCEs from summer 2016 (*M* = 3.33, SD = .79) to winter 2017 (*M* = 3.98, SD = .56) (*t* (11)= − 3.184, *p* < 0.01). Further, intraclass correlation coefficient calculations demonstrated moderate agreement between in-person and blind ratings of resident entrustment on the four OSCE cases (see Table 2). The agreements were statistically significant (*p* < 0.05).

**Table 2:** OSCE performance assessment

OSCE case	Mean entrustment scores (SD)		*α*	ICC	*p* value
	Live	Blind			
1	3.43 (.756)	3.46 (1.127)	0.636	0.653	0.047*
2	3.57 (.756)	4.07 (1.072)	0.654	0.609	0.033*
3	3.87 (.834)	2.93 (.829)	0.802	0.609	0.003*
4	4.07 (.704)	3.40 (.828)	0.717	0.585	0.013*

*OSCE* objective structured clinical examination, *SD* standard deviation, *α* Cronbach’s alpha, *ICC* intraclass correlation coefficient; *statistical significance

Different themes emerged from the workplace-based narrative and the simulation-based narrative comments, indicating that the different settings prompted different feedback for the learners and that some difference may have existed in the competencies assessed. Themes emerging from the workplace-based narrative feedback included a focus on overall performance, general medical management, leadership, and interaction with others in the ED (i.e., communication with nurses, communication with family, supervision and teaching of more junior learners, interaction with consultants), as indicated in Table 3. In contrast, simulation-based narrative comments focused more on task-specific feedback and details in medical management (see Table 4). Both sets of data included commentary on communication skills, with communication being one of the most frequently used words in both narrative data sets.

**Table 3:** Thematic analysis of workplace-based RAT narrative comments

Workplace-based RAT narrative	
Themes	Examples
Interaction with others in the ED	“Managed both resus cases well: supervised and directed junior resident and resuscitation; stayed in charge with nursing/staff.”
Overall performance	“Excellent problem solving of a difficult cardiac arrest. Good situational awareness.”
Leadership	“Led team. Delegated responsibility. Shared mental model clearly particularly when time to call the arrest.”
General medical management	“Very capable resuscitation including acute airway management with appropriate technique … Provided him some minor coaching only regarding choice of drugs.”
Communication	“Well done. Calm, controlled manner. … Communication by phone by POA. Consulted ICU.”

*RAT* resuscitation assessment tool

**Table 4:** Thematic analysis of simulation-based RAT narrative comments

Simulation-based RAT narrative	
Themes	Examples
Task-specific feedback	“Earlier pacing! Early recognition of probable hyperkalemia.” “Reasonably quick pacing. No need to sync. ECG done—needs more interpretation quickly.”
Details in medical management	“Good handling of glucose distraction, airway equipment, allergies!” “Pacing fair – 25mcg fentanyl inadequate”
Communication	“Excellent communication …” “Good delegation of actions, good communication to team re: (treatment and) thoughts …”

*RAT* resuscitation assessment tool

## Discussion

Our findings suggest that residents’ resuscitation performance in a simulated setting approximates their resuscitation performance in the clinical workplace. However, as expected, this positive relationship is imperfect and speaks to the challenges with workplace-based assessment in general. Primarily, the comparison of workplace-based assessment and simulation-based assessments may not have been comparing “apples to apples”. There was no controlling for specific clinical cases assessed in the workplace beyond attending physician categorization of resuscitation and resident choice. It is entirely possible that trainees assessed on a limited number of cases in the workplace were assessed on very different clinical content than in the simulation lab (see Table 1) and therefore had variable performance across domains due to differences in competence in managing specific case presentations. Moreover, the workplace-based assessment was primarily a resident-driven tool and may have been biased in the selection of cases to reflect more favorably on the learner than if it had been faculty-driven like the OSCE. Indeed, our data does show a trend of increased mean entrustment scores in the workplace-based setting (4.19) compared to the simulation lab (3.34). Furthermore, the workplace-based assessment was seen as the gold standard in this study and is a standard that is fraught with bias [37]. Simulation performance may actually better reflect learner competence on specific resuscitation skills with the extraneous and uncontrollable environment of the real-world ED taken away, especially if assessors can more closely focus on residents’ medical management and not on patient care. Regardless of its associated challenges, performance in the workplace is ultimately the endpoint of interest in the training of competent physicians and thus was chosen as the comparator.

Our qualitative findings suggest that in making entrustment decisions in the simulation and clinical environments, faculty may be focusing on different aspects of performance. This finding presents an intriguing starting point for further investigation. In the workplace, assessors commented on how residents’ generally function within the resuscitation environment, including how they engage in medical management, communicate with others, and lead a team. However, in the simulation setting, assessors used the RAT to provide brief, task-specific feedback with more point form notes on medical management and communication. The complex environment of the ED and the priority of patient care make a careful direct observation in resuscitation and immediate feedback difficult for assessors in the workplace. In contrast, the simulation lab is controlled, has fewer unplanned distractors, and has dedicated time for a thorough debrief and targeted feedback. In this way, the simulation lab is more conducive to feedback on specific details of medical management than the workplace. Although staff were encouraged to complete assessments on trainees immediately following resuscitations, this was not consistently done. Though practically more feasible, the practice of delayed assessment may have the potential to encourage the generation of broad reflections on performance as opposed specific-targeted feedback relevant to aspects of the resuscitation case itself.

In this new climate of decreased duty hours, improved patient safety, social accountability, and de-emphasis on time-based accomplishments, there is a need for novel ways to objectively and reliably assess our learners’ performance of complex competencies [38]. Assessment in a simulation environment is a structured, predictive, and comprehensive method to evaluate clinical performance [39]. The ED, in contrast, is limited and opportunistic in nature, with many competing interests beyond learner improvement, most importantly patient safety. Taking this further, simulation can be thought of not only as a tool for frequent formative assessments, but also potentially as a high-stakes summative assessment tool [40]. Several organizations have embraced simulation as a summative and high-stakes assessment opportunity, such as the American Board of Anesthesiology [41], the Israeli Board of Anesthesia [42], Ornge (formerly Ontario Air Ambulance Corporation) [43], and the Canadian National Anesthesiology Simulation Curriculum [44].

In the new era of CBME, assessment of resuscitation performance in a simulated environment can contribute meaningful performance information to a comprehensive program of assessment. Incorporation of simulation in programmatic assessment allows learners to be assessed on complex aspects of patient care without clinical consequence and to learn through the process of receiving feedback for improvement. However, the imperfect correlation and different focus of feedback in simulation and clinical environments suggest that using one without the other may lead to missing data in the complete picture of resident competency assessment. Taken together, these findings highlight the importance of triangulating quantitative and qualitative evidence of resuscitation performance across simulation and real-life clinical settings to look for patterns and discrepancies across contexts.

## Limitations

Despite providing some preliminary evidence for the expanded use of simulation in resuscitation assessment, there are noteworthy limitations which deserve mention in our study. Primarily, the lack of complete data sets collected for each resident, and the resulting small sample size, limits the significance and generalizability of our results. Only 64% of our resident cohort had data that was sufficient to analyze, with an inconsistent number of RATs (between one and nine) completed for each individual resident. This may be due to the scheduling issues (with many residents away on rotation), preferential utilization of the RAT by senior residents in the workplace, illness, and other conflicts. We argue that many of these factors, while resulting in a reduced sample size, did not systematically bias the sample of assessment data in a way that would alter the results in a specific direction. The small sample size certainly may have resulted in either a dilution of correlation or a falsely stronger correlation by chance, and as such, the generalizability of our results should not be overstated. Ultimately though, while the low number of participants in this study is a limitation, a plausible signal persists and is worthy of discussion.

The year-long timeline of the project, and subsequent resident progression in skillset and competence, may have affected the comparison. Residents displayed improvement on simulation OSCE performance from August 2016 to February 2017. New residents to the training program enter with variable experience with simulation, which may have resulted in a stronger influence of environment unfamiliarity on resident performance in the simulation environment. Ideally, the workplace-based assessments and the simulation-based assessments would be temporally matched to control for any learning that inevitably occurs throughout a year of residency training. This was not done in the present study. Despite this, the positive correlation between simulation performance and real-world performance persisted and likely represents a realistic assessment of a dynamic target.

Beyond the data points obtained, the nature of the data collected carries with it an inherent bias well recognized in the literature with unblinded assessors (e.g., the halo effect) [45]. This being said, blinded external raters were used in the simulation setting as a check and were found to have moderate agreement with unblinded raters using intraclass correlation coefficients. The difference in rating by blind external raters and local in-person rating can be attributed to multiple factors including the abovementioned halo effect, leniency bias, interpersonal relationships with the trainee, and preceding experience with the trainee. Unfortunately, blinded rating was not possible in the real-world setting due to logistical and ethical constraints. Additionally, all front-line faculty had the opportunity to be an assessor in the real-world setting, but only a selected group of faculty completed simulation-based assessments. This may have introduced increased variability in assessment scoring.

Lastly, while the RAT was based on the previously studied and evaluated QSAT, there is limited validity evidence available specifically supporting the RAT. Here, we suggest that the strong body of evidence supporting the original QSAT in simulation-based OSCEs [14, 27, 28] combined with a groundswell of support for the utilized entrustment score [30] and correlation between the entrustment score and the QSAT global assessment score [31] combine to argue for the validity of the RAT. Future work evaluating the RAT specifically needs to be done.

## Conclusion

This study demonstrates that among EM residents at a single training site, assessment of resuscitation performance in a simulated setting approximates assessment of resuscitation performance in the clinical workplace on non-matched case presentations. This study was limited by a low sample size; future studies with larger sample sizes and across multiple centers are needed to provide further extrapolation evidence to support the validity of simulation-based assessment of resuscitation competence.

## Additional file

Additional file 1: Queen’s simulation assessment tool (QSAT). (DOCX 128 kb)

## Abbreviations

CBME: Competency-based medical education
ED: Emergency department
EM: Emergency medicine
OSCE: Objective structured clinical exam
PGY: Postgraduate year
QSAT: Queen’s simulation assessment tool
RAT: Resuscitation assessment tool

## References

[1] Ten Cate O, Hart D, Ankel F (2016). Entrustment decision making in clinical training. Acad Med.

[2] Isaak RS, Chen F, Martinelli SM (2018). Validity of simulation-based assessment for accreditation council for graduate medical education milestone achievement. Simul Healthc J Soc Simul Healthc..

[3] Cook DA, Brydges R, Zendejas B, Hamstra SJ, Hatala R (2013). Technology-enhanced simulation to assess health professionals: a systematic review of validity evidence, research methods, and reporting quality. Acad Med.

[4] Brydges R, Hatala R, Zendejas B, Erwin PJ, Cook DA (2015). Linking simulation-based educational assessments and patient-related outcomes. Acad Med.

[5] Cook DA, Zendejas B, Hamstra SJ, Hatala R, Brydges R (2014). What counts as validity evidence? Examples and prevalence in a systematic review of simulation-based assessment. Adv Heal Sci Educ.

[6] Okuda Y, Bryson EO, Jr SD, Quinones J, Shen B, Levine AI. The utility of simulation in medical education : what is the evidence ? Mt Sinai J Med. 2009;(76):330–43. 10.1002/MSJ. 10.1002/msj.20127 19642147

[7] McGaghie WC, Issenburgh SCCE e a (2011). Does simulation based medical education yield better results than traditional clinical education? A meta-analytic comparative review of the evidence. Acadm Meded.

[8] Ahmed K, Jawad M, Abboudi M (2011). Effectiveness of procedural simulation in urology : a systematic review. J Urol.

[9] Bohnen Jordan D., Demetri Leah, Fuentes Eva, Butler Kathryn, Askari Reza, Anand Rahul J., Petrusa Emil, Kaafarani Haytham M.A., Yeh D. Dante, Saillant Noelle, King David, Briggs Susan, Velmahos George C., Moya Marc de (2018). High-Fidelity Emergency Department Thoracotomy Simulator With Beating-Heart Technology and OSATS Tool Improves Trainee Confidence and Distinguishes Level of Skill. Journal of Surgical Education.

[10] Zendejas B, Brydges R, Wang AT, Cook DA (2013). Patient outcomes in simulation-based medical education: a systematic review. J Gen Intern Med.

[11] Manser T, Dieckmann P, Wehner T, Rall M (2007). Comparison of anaesthetists’ activity patterns in the operating room and during simulation. Ergonomics..

[12] Couto TB, Kerrey BT, Taylor RG, FitzGerald M, Geis GL (2015). Teamwork skills in actual, in situ, and in-center pediatric emergencies. Simul Healthc J Soc Simul Healthc.

[13] Savoldelli GL, Naik VN, Joo HS (2006). Evaluation of patient simulator performance as an adjunct to the oral examination for senior anesthesia residents. Anesthesiology..

[14] Hall Andrew Koch, Damon Dagnone J., Moore Sean, Woolfrey Karen G. H., Ross John A., McNeil Gordon, Hagel Carly, Davison Colleen, Sebok-Syer Stefanie S. (2017). Comparison of Simulation-based Resuscitation Performance Assessments With In-training Evaluation Reports in Emergency Medicine Residents: A Canadian Multicenter Study. AEM Education and Training.

[15] Ghaderi I, Vaillancourt M, Sroka G (2011). Performance of simulated laparoscopic incisional hernia repair correlates with operating room performance. Am J Surg.

[16] McCluney AL, Vassiliou MC, Kaneva PA (2007). FLS simulator performance predicts intraoperative laparoscopic skill. Surg Endosc Other Interv Tech.

[17] Datta V, Bann S, Beard J, Mandalia M, Darzi A (2004). Comparison of bench test evaluations of surgical skill with live operating performance assessments. J Am Coll Surg.

[18] Wilasrusmee C, Lertsithichai P, Kittur DS (2007). Vascular anastomosis model: relation between competency in a laboratory-based model and surgical competency. Eur J Vasc Endovasc Surg.

[19] Miller GE (1990). The assessment of clinical skills/ competence/performance. AAMC Acad Med J Assoc Am Med Coll.

[20] Boursicot K, Etheridge L, Setna Z (2011). Performance in assessment: consensus statement and recommendations from the Ottawa conference. Med Teach.

[21] Amin Z, Boulet JR, Cook DA (2011). Technology-enabled assessment of health professions education: consensus statement and recommendations from the Ottawa 2010 conference. Med Teach..

[22] Hall AK, Pickett W, Dagnone JD (2012). Development and evaluation of a simulation-based resuscitation scenario assessment tool for emergency medicine residents. Can J Emerg Med..

[23] Weller JM, Misur M, Nicolson S (2014). Can i leave the theatre? A key to more reliable workplace-based assessment. Br J Anaesth.

[24] Ten Cate O (2016). Entrustment as assessment: recognizing the ability, the right, and the duty to act. J Grad Med Educ.

[25] Cook DA, Brydges R, Ginsburg S, Hatala R (2015). A contemporary approach to validity arguments: a practical guide to Kane’s framework. Med Educ.

[26] Hatala R, Cook DA, Brydges R, Hawkins R (2015). Constructing a validity argument for the objective structured assessment of technical skills (OSATS): a systematic review of validity evidence. Adv Heal Sci Educ..

[27] Hall AK, Dagnone JD, Lacroix L, Pickett W, Klinger DA (2015). Queen’s simulation assessment tool: development and validation of an assessment tool for resuscitation objective structured clinical examination stations in emergency medicine. Simul Healthc.

[28] Dagnone JD, Hall AK, Sebok-Syer S (2016). Competency-based simulation assessment of resuscitation skills in emergency medicine postgraduate trainees - a Canadian multi-centred study. Can Med Educ J.

[29] Hsu C, Sandford B (2007). The delphi technique: making sense of consensus. Pract Assessment, Res Eval.

[30] Ten Cate O, Hart D, Ankel F (2015). Entrustment decision making in clinical training. Acad Med.

[31] Hagel C, Hall AK, Klinger D, McNeil G, Dagnone JD (2016). P057: performance of a national simulation-based resuscitation OSCE for emergency medicine trainees. Can J Emerg Med.

[32] Ten Cate O, Chen HC, Hoff RG, Peters H, Bok H, Van Der Schaaf M (2015). Curriculum development for the workplace using Entrustable Professional Activities (EPAs): AMEE guide no. 99. Med Teach..

[33] Committee E and P. Education and prevention committee interpretive bulletin. Vol 8.; 2009. https://www.oma.org/wp-content/uploads/0804epc_bulletin.pdf.

[34] Dagnone JD, McGraw R, Howes D (2016). How we developed a comprehensive resuscitation-based simulation curriculum in emergency medicine. Med Teach..

[35] Hagel CM, Hall AK, Damon Dagnone J (2016). Queen’s university emergency medicine simulation osce: an advance in competency-based assessment. Can J Emerg Med..

[36] Attride-stirling J (2001). Thematic networks: an analytic tool for qualitative research. Qual Res.

[37] Govaerts MJB, Van de Wiel MWJ, Schuwirth LWT, Van der Vleuten CPM, Muijtjens AMM (2013). Workplace-based assessment: raters’ performance theories and constructs. Adv Heal Sci Educ..

[38] Harris P, Bhanji F, Topps M (2017). Evolving concepts of assessment in a competency-based world. Med Teach..

[39] Cook DA, Brydges R, Zendejas B, Hamstra SJ, Hatala R (2013). Mastery learning for health professionals using technology-enhanced simulation. Acad Med.

[40] Boulet JR (2008). Summative assessment in medicine: the promise of simulation for high-stakes evaluation. Acad Emerg Med.

[41] Steadman RH, Huang YM (2012). Simulation for quality assurance in training, credentialing and maintenance of certification. Best Pract Res Clin Anaesthesiol.

[42] Berkenstadt H, Ziv A, Gafni N, Sidi A (2006). Incorporating simulation-based objective structured clinical examination into the Israeli national board examination in anesthesiology. Anesth Analg.

[43] Tavares W, LeBlanc VR, Mausz J, Sun V, Eva KW (2014). Simulation-based assessment of paramedics and performance in real clinical contexts. Prehospital Emerg Care.

[44] Chiu M, Tarshis J, Antoniou A (2016). Simulation-based assessment of anesthesiology residents’ competence: development and implementation of the Canadian National Anesthesiology Simulation Curriculum (CanNASC). Can J Anesth.

[45] Sherbino J, Norman G (2017). On rating angels: the halo effect and straight line scoring. J Grad Med Educ..

